# Shenfu injection promotes self-renewal of intestinal stem cells in sepsis-induced intestinal injury via inducing ATF4 expression

**DOI:** 10.3389/fphar.2025.1500157

**Published:** 2025-07-31

**Authors:** Yunxin Deng, Lingyun Lu, Jinrong Li, Liya Gao, Yihui Wang, Nataša Petrović, Fengyong Yang, You Li, Mei Meng

**Affiliations:** ^1^ Department of Critical Care Medicine, Shanghai Ninth People’s Hospital, Shanghai Jiao Tong University School of Medicine, Shanghai, China; ^2^ Department of Traditional Chinese Medicine, Shanghai Jiao Tong University School of Medicine Affiliated Ruijin Hospital, Shanghai, China; ^3^ Yantai Affiliated Hospital of Binzhou Medical University, Binzhou, Shandong, China; ^4^ Department of Emergency, Ruijin Hospital, Shanghai Jiao Tong University School of Medicine, Shanghai, China; ^5^ Department of Anesthesia of Urology Clinic, Centre of Anesthesiology and Reanimatology, University Clinical Centre of Serbia, Belgrade, Serbia; ^6^ Jinan People’s Hospital Affiliated to Shandong First Medical University, Jinan, China; ^7^ Department of Surgery, Ruijin Hospital, Shanghai Jiao Tong University School of Medicine, Shanghai, China

**Keywords:** Shenfu injection, sepsis, intestinal dysfunction, crypt stem cell, gut microbiota

## Abstract

**Introduction:**

Shenfu injection (SFI) is widely used in clinical severe conditions including sepsis due to its pharmacological effects of invigorating Qi and reviving Yang for resuscitation. SFI is known to alleviate sepsis-related intestinal injury. However, the underlying mechanism remains exclusive. This study aimed to investigate the regulatory role of SFI in the self-renewal of intestinal stem cells as well as gut microbiota during sepsis.

**Methods:**

Plasma of septic patients was collected for detecting intestinal dysfunction markers. Mechanistic investigations were performed using immunofluorescent co-staining, Western Blotting, hematoxylin-eosin(H&E) staining, immunohistochemical (IHC) staining, TUNEL staining, Enzyme-linked immunosorbent assay (ELISA), quantitative reverse transcription-PCR (RT-qPCR) and 16S rRNA gene sequencing.

**Results:**

Plasma levels of D-lactate and intestinal-type fatty acid-binding protein (I-FABP) in septic patients with intestinal dysfunction were significantly reduced following SFI administration. Compared to the control mice, sepsis induced severe mucosal damage in the intestine, including the decrease of the villus length and the number of crypts, which was significantly inhibited by SFI administration. In addition, SFI significantly reduced the levels of pro-inflammatory cytokines Interleukin 1β (IL-1β), Interleukin 6 (IL-6), and tumor necrosis factor-α (TNF-α) in septic mice. Cell proliferation and migration in the intestine were significantly increased in the intestine of septic mice upon SFI treatment. Mechanically, SFI facilitated the expression of activating transcription factor 4 (ATF4) to promote the transcription of SRY-Box Transcription Factor 9 (Sox9), thereby activating the stemness and proliferation of crypt stem cells. Moreover, the gut microbiota composition in septic mice was modulated by SFI administration. In particular, high dose of SFI (HSF) treatment was found to decrease the proportion of harmful bacteria including Proteobacteria and Escherichia-Shigella, while increase the abundance of beneficial bacteria such as Alloprevotella and Butyricimonas in septic mice.

**Discussion:**

Our findings revealed that SFI protects against sepsis-induced intestinal injury via promoting the self-renewal of crypt stem cells and regulating the gut microbiota composition, providing strong evidence for the potential of SFI in treating sepsis-related intestinal dysfunction.

## 1 Introduction

Sepsis is a life-threatening condition characterized by infection-induced dysregulation of immune responses as well as multiple organ dysfunction syndrome (MODS). Despite of advanced supportive medical strategies, sepsis remains the leading cause of death in the intensive care unit. The gut system is easily and frequently disrupted during sepsis and therefore serves as the motor of MODS (Mittal and Coopersmith, 2014). In sepsis, the integrity of the gastrointestinal tract is compromised by increased cellular apoptosis and altered barrier permeability, contributing to the development of bacterial translocation and systemic infection, thereby aggravating sepsis severity (Wang C. et al., 2019). The intestinal epithelium undergoes continual renewal from multipotent stem cells at the base of the intestinal crypts (Guo et al., 2023). Crypt stem cells proliferate and differentiate into multiple distinct epithelial subtypes which migrate upward to the villus tips to maintain intestinal integrity (Barker et al., 2007; Parker et al., 2017). We previously reported that sepsis reduces the proliferation of crypts and slows the migration of enterocytes, implying that intestinal regeneration is a potential target for sepsis-related intestinal injury (Meng et al., 2019).

Shenfu injection (SFI) is a modern traditional Chinese medicine derived from Shenfu decoction. SFI is a water solution extracted from Panax ginseng C. A. Mey and Aconitum carmichaeli Debeaux (Li et al., 2015). Ginsenosides and aconitum alkaloids are the main active constituents of SFI (Ge et al., 2015). Given the pharmacological effects of invigorating Qi and reviving Yang for resuscitation, SFI has been widely applied in severe clinical conditions, such as shock, heart failure, myocardial ischemia reperfusion injury, and cerebrovascular diseases (Zhang and Li, 2023; Xu et al., 2020). Increased evidences have reported the benefits of SFI in sepsis-related intestinal dysfunction (Wang et al., 2022; Luo et al., 2021). However, the underlying mechanisms remains unclear.

In this study, we discovered for the first time that SFI could increase the proliferation of crypt stem cells via activating transcription factor 4 (ATF4)-mediated expression of the stemness marker SRY-Box Transcription Factor 9 (Sox9), thereby facilitating the self-repair of the villus. In addition, the downregulations of the tight junction protein Zonula Occludens-1 (ZO-1) and Occludin were significantly restored upon SFI administration. Apoptosis in the intestinal tissues was effectively reduced by SFI treatment. Moreover, high dose of SFI (HSF) treatment decreased the proportion of harmful bacteria, including Proteobacteria and Escherichia-Shigella, while increasing the abundance of beneficial bacteria such as Alloprevotella and Butyricimonas in septic mice. Our findings revealed the beneficial effects of SFI on the self-renewal of crypt stem cells in sepsis-related intestinal injury, supporting the clinical benefits of SFI in treating sepsis-associated intestinal barrier dysfunction.

## 2 Materials and methods

### 2.1 Patients

Patients diagnosed with sepsis in the Department of Critical Care Medicine in Shanghai Ruijin Hospital from October 1, to 31 May, 2024, were prospectively enrolled. This study protocol conformed to the ethics guidelines of the Declaration of Helsinki and was approved by the ethics committee of Ruijin Hospital (No. 20210101). Informed consent was obtained from each participant.

The enrollment criteria were as follows: (1) patients aged 18–90 years, (2) meeting sepsis 3.0 definition, and (3) hospital stay >24 h. Exclusion criteria were: (1) discharge or death within 24 h after admission; (2) participation in other clinical research; (3) emergency surgery after admission; (4) malignant tumor; (5) pregnant or lactating patients; (6) lack of necessary clinical data; and (7) allergy to SFI or the ingredients of SFI. The SFI was administered to the patients according to the decision of the attending physician. Finally, 41 patients with sepsis were enrolled, including 20 patients who used SFI (50 mL continuous intravenous infusion every 6 hours for 5 days) while 21 patients who did not.

Peripheral blood was withdrawn from patients with or without SFI treatment on the fifth day of diagnosis. Plasma samples were obtained after centrifugation at 2,000 rpm for 10 min and were stored at −80°C for further analysis (Supplementary Table S1). D-lactate plasma concentration was measured by detecting absorbance at 450 nm following the manufacturer’s introductions (D-Lactate Assay Kit, Abcam, ab83429).

### 2.2 Shenfu injection

Shenfu injection (SFI, batch number: 221101AK01) was obtained from Huarun Sanjiu Pharmaceutical Co., Ltd. (Ya’an, Sichuan, P.R. China). Its quality was analyzed using fingerprint technology during production and was controlled according to the standards of the China Food and Drug Administration (WS3-B-3427-98-2013 and P. ZL. 205-001) (Table 1). The voucher specimens were stored at the herbarium center of Huarun Sanjiu Pharmaceutical Co., Ltd. (Panax ginseng C.A. Mey: Voucher No. A17201208001, Ratio 47%; Aconitum carmichaelii Debeaux: Voucher No. A17220921003, Ratio 17.1%).

**TABLE 1:** The quality control analysis of SFI (batch number: 221101AK01).

Item	Chemical structure	Content	Quality control
Fingerprint consistency		0.9	≥0.9
Total saponin		1.4 mg/mL	0.7–1.7 mg/mL
Ginsenoside Rg1	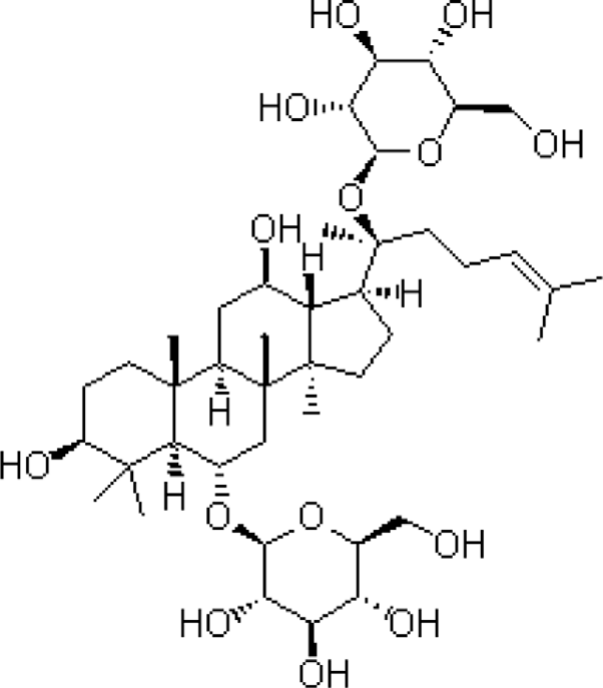	0.11 mg/mL	≥0.08 mg/mL
Ginsenoside Re	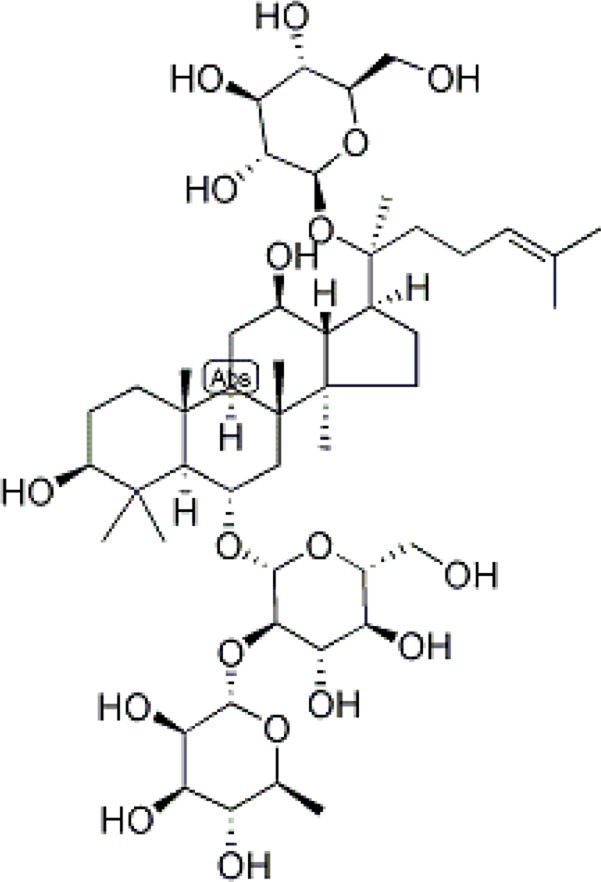	0.08 mg/mL	≥0.06 mg/mL

### 2.3 Animals

Male C57BL/6J mice (8–10 weeks, 20–25 g) were obtained from Charles River (Beijing, China) and randomly divided into experimental groups (n = 6 per group). Before induce sepsis, mice were intraperitoneally injected with pentobarbital (New Asia Pharma, Shanghai, China) for anesthesia (50 mg/kg). Sepsis was induced by surgically performing cecal ligation and puncture (CLP). Briefly, after anesthesia, the abdominal wall was incised for 2–3 cm. The cecum was carefully exposed and ligated with a 5–0 silk thread 15 mm from the apex and passed through the distal part of the cecum using a 20-gauge needle. After removing a small amount of faecal content, the cecum was reset into the abdominal cavity and the exposed abdominal wall was closed. Mice in the sham group underwent the same procedure but without cecum ligation and puncture. All mice were rehydrated with 1 mL 0.9% saline subcutaneous injection at the end of surgery and were free to move around to obtain water and food. In the intervention groups, mice were intraperitoneally injected with a low dose of SFI (LSF) (10 mL/kg) and HSF (20 mL/kg) 1 h after CLP surgery. 48 h after CLP challenge, all mice were anesthetized with the animal anesthetic pentobarbital via intraperitoneal injection at a dose of 125 mg/kg (Pang and Laferriere, 2020). After the depth of anesthesia is ensured the mice were rapidly euthanized by spinal dislocation method to collect tissue. Fecal samples close to the anus were collected for microbiome analysis. Blood samples were collected from retro-orbital sinus for further analysis. For proliferation and migration analysis, 5-Ethynyl-2′- deoxyuridine (EdU) was i.p. injected before CLP challenge or 90 min before the mice were sacrificed. The intestines were collected, cleaned, and fixed in 4% formalin for further examination.

### 2.4 Immunofluorescent co-staining of EdU/olfactomedin 4 (OLFM4)/epithelial cellular adhesion molecule (EpCAM)

After deparaffinization and dehydration, intestinal sections were incubated in Ethylenediaminetetraacetic acid (EDTA) (PH 9.0) for 1 min 30 s for antigen retrieval, and then blocked with 10% normal goat serum for 1 h at room temperature. Sections were incubated with antibodies against EpCAM (ab221552, Abcam, Cambridge, United Kingdom) at 4°C overnight. Then sections were incubated with horseradish peroxidase (HRP)-conjugated Goat Anti-rabbit secondary antibody for 1 h at 37°C. After washing, sections were incubated with Fluorescein isothiocyanate (FITC) solution for 10 min at room temperature. After washing, the sections were incubated in citric acid (PH 6.0) for 5 min. After blocking with 10% normal goat serum for 20 min at room temperature, sections were incubated with OLFM4 (39141, CST, Danvers, MA, United States) at 4°C overnight. Then sections were incubated with HRP-conjugated Goat Anti-rabbit secondary antibody for 1 h at 37°C. After washing, sections were incubated with Cyanine3 (Cy3) for 10 min at room temperature. After washing, sections were stained with EdU in Click-iT EdU Reaction Buffer (Thermo Fisher Scientific). Nuclei were stained with 4,6-diamidino-2-phenylindole (DAPI) for 5 min in the dark at room temperature, and then stained with diaminobenzidine for microscopic examination (DS-U3; Nikon, Tokyo, Japan). The slides were mounted with mounting media for further scanning (Sigma, Missouri, United States).

### 2.5 Immunofluorescent co-staining of ATF4/OLFM4

After deparaffinization and dehydration, intestinal sections were incubated in EDTA (PH 9.0) for 1 min 30 s for antigen retrieval and then blocked with 10% normal goat serum for 1 h at room temperature. Sections were incubated with antibodies against ATF4 (11815, CST) and OLFM4 at 4°C overnight. The sections were then incubated with HRP-conjugated Goat Anti-rabbit secondary antibody for 1 h at 37°C. After washing, sections were incubated with Cy3 for 10 min at room temperature. Nuclei were stained with DAPI for 5 min in the dark at room temperature, and then stained with diaminobenzidine for microscopic examination (DS-U3; Nikon, Tokyo, Japan). The slides were mounted with mounting media for further scanning (Sigma, Missouri, United States).

### 2.6 Western blotting

Mouse intestinal tissues were lysed using RIPA buffer (#89901, Thermo fisher Scientific) supplemented with PhosSTOP and complete protease inhibitors (Merck, Beijing, China). Protein samples were subjected to 12.5% SDS-PAGE and transferred to a polyvinylidene fluoride membrane (162-0177, BioRad, Shanghai, China). Blots were probed with appropriate primary antibodies against ATF4 (11815, CST), Sox9 (3579, CST), OLFM4 (39141, CST), and phosphorylated extracellular signal-regulated kinases 1 and 2 (ERK1/2) (9101, CST) at 4°C overnight. The blots were then incubated with the appropriate secondary antibodies (1:5000, Proteintech) and detected using HRP substrate (WBLUF0500, Millipore, United States). For internal controls of equal loading, the blots were stripped with stripping buffer (100 mmol/L 2-mercaptoethanol, 2% SDS, 62.5 mmol/L, Tris pH 6.8) and re-probed with ERK1/2 or Glyceraldehyde-3-Phosphate Dehydrogenase (GAPDH) antibodies.

### 2.7 Hematoxylin-eosin (H&E) staining

Paraffin blocks of intestinal tissues were cut into sections with a thickness of 5 µm. After deparaffinization and hydration, the sections were stained with hematoxylin and eosin solution. H&E images were obtained randomly from the stained sections. Pathological scores were analyzed based on histopathological criteria for intestinal damage (Chen et al., 2021).

### 2.8 Immunohistochemical (IHC) staining

Mouse intestinal tissue samples were fixed in 4% formalin and embedded in Paraffin. After deparaffinization and dehydration, sections were antigen retrieved and incubated with ZO-1 (ab221547, Abcam), Occludin antibodies (ab216327, Abcam), and cleaved-caspase 3 (9664, Cell Signaling Technology) at 4°C overnight, and then incubated with biotinylated secondary antibody at 37°C for 30 min. Finally, the sections were visualized using 3,3′-diaminobenzidine (DAB) solution and counterstained with hematoxylin. Images were captured using a light microscope (BX50, Olympus). IHC staining was analyzed and quantified by pathologists.

### 2.9 Tunnel staining

Mouse intestinal tissue samples were fixed in 4% formalin and embedded in Paraffin. After deparaffinization and dehydration, the sections were incubated with Proteinase K (20 μg/mL, A113-03, Vazyme, Jiangsu, China) for 10 min at room temperature. After washing, sections were incubated with 1xEquilibration buffer for 30 min at room temperature. Discard 1xEquilibration buffer on the sections and incubate sections with recombinant TdT enzyme (Vazyme) for 60 min at 37°C in dark. After washing, the sections were stained with DAPI solution (1:500) for 5 min at room temperature in the dark. Finally, the sections were mounted and scanned.

### 2.10 Enzyme-linked immunosorbent assay (ELISA)

The plasma levels of intestinal-type fatty acid-binding protein (I-FABP) in septic patients were measured using an ELISA kit (CSB-E08024h, CUSABIO Technology, Wuhan, China). Lactate levels in mouse plasma were detected using a lactate assay kit according to the manufacturer’s instructions (L-Lactate Assay Kit, 1200011002, Eton Bioscience). The absorbance was measured at 490 nm. The levels of diamine oxidase (DAO) in mouse plasma were measured using a DAO assay kit (E-EL-M0412c, Elabscience, Shanghai, China) according to the manufacturer’s instructions.

### 2.11 RNA extraction and quantitative RT-PCR

Total RNA was extracted from intestinal tissue lysates using the TRIzol reagent (R401, Vazyme, Jiangsu, China) following the manufacturer’s protocol. The RNA concentration and purity were measured using a Gen5 Microplate Spectrophotometer (BioTek, VT, United States). Reverse transcription (RT) of RNA was performed using the HiScript III RT SuperMix (R323, Vazyme). ChamQ Universal SYBR qPCR Master Mix (Q711, Vazyme) was used for quantitative polymerase chain reaction to analyze the mRNA levels.

### 2.12 16S rRNA gene sequencing

Sequencing of the cecal microbiota was performed on an Illumina NovaSeq6000 (Illumina Inc., San Diego, CA, United States) and 250 bp paired-end reads were generated. Raw sequencing data were provided in the FASTQ format. Cutadapt software was used to remove sequences without primers and cut off the adapter. Uparse v7.0.1001 was used to obtain operational taxonomic units (OTUs) for species annotation and microbial diversity analysis. Alpha diversity was analyzed using the Chao1, Shannon, and Simpson indices. Beta diversity was displayed using unweighted UniFrac Non-metric Multidimensional Scaling (NMDS) analysis. The 16S rRNA gene sequencing and relevant data analysis were conducted by OE Biotech Co., Ltd. (Shanghai, China).

### 2.13 Statistical analysis

All data are expressed as mean ± standard deviations (SD). Statistical analysis was performed using one-way analysis of variance (ANOVA) followed by Bonferroni’s multiple comparisons test. Figures were prepared using GraphPad Prism version 9.0 (GraphPad Software, San Diego, CA, United States).

## 3 Results

### 3.1 Shenfu injection alleviated the intestinal injury in sepsis

**FIGURE 1:**
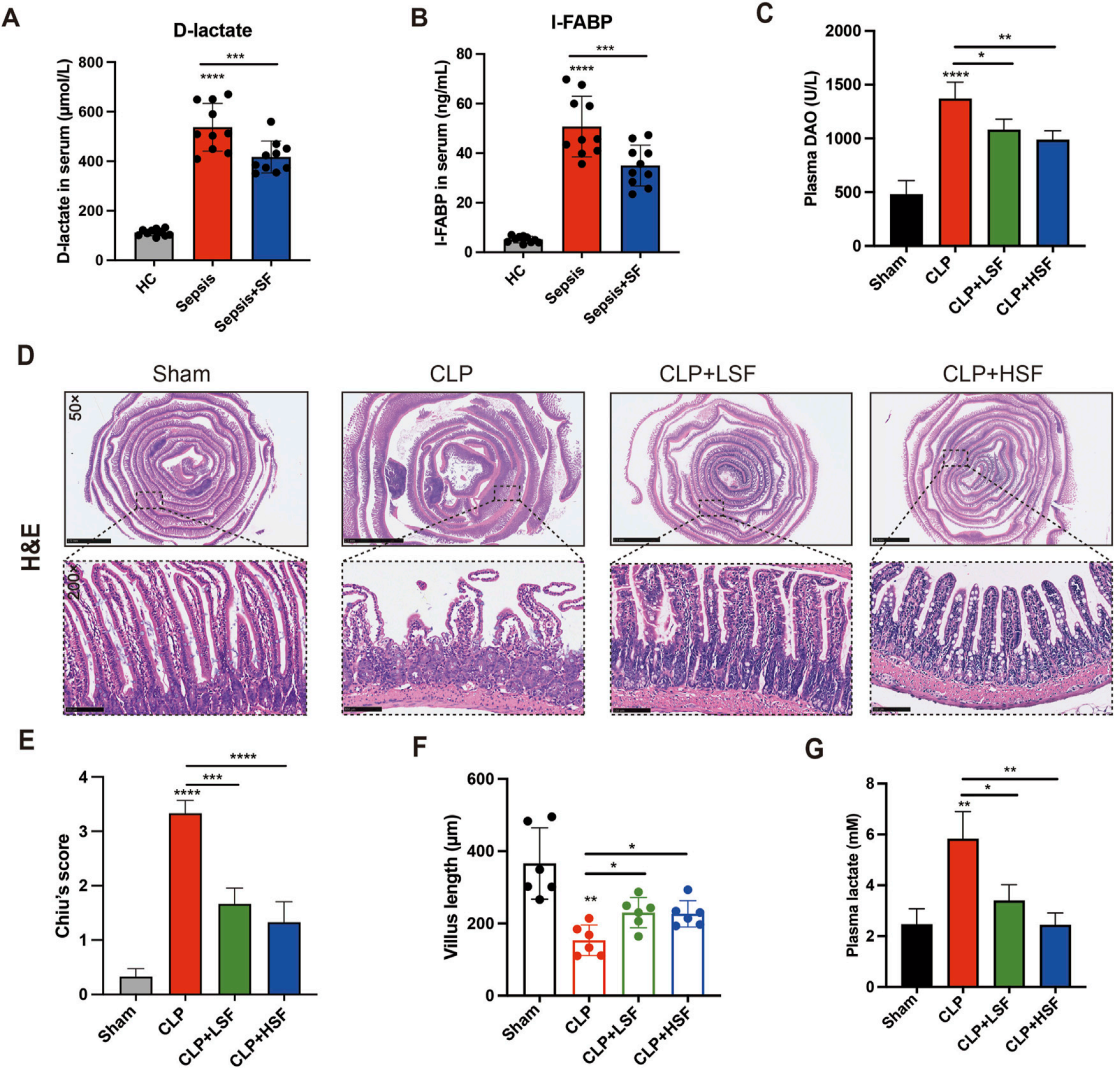
SFI administration alleviated sepsis-induced intestinal injury. **(A,B)** The plasma levels of D-lactate and I-FABP in septic patients were measured (n = 10). **(C)** The plasma levels of DAO in septic mice treated with or without LSF and HSF were measured (n = 6). **(D)** H&E staining of the intestinal tissues. **(E)** Pathological evaluation of the intestinal injury using Chiu’s score (n = 6 per group). **(F)** The length of the villus in selected fields were measured (n = 6 per group). **(G)** The levels of lactate in the plasma were measured by lactate assay kit. *, P < 0.05, **, P < 0.01, ***, P < 0.001, ****, P < 0.0001.

The clinical data showed that the plasma levels of D-lactate and I-FABP in septic patients with intestinal dysfunction were significantly reduced upon SFI administration (Figures 1A,B). No SFI-related adverse events had been reported in our study. To evaluate the effects of SFI on intestinal injury during sepsis, mice were challenged with CLP and treated with different doses of SFI (10 mL/kg and 20 mL/kg). The plasma levels of DAO were significantly higher in the septic group than in the control group. SFI treatment significantly decreased the levels of DAO (Figure 1C). The H&E staining showed that the intestinal mucosal structures were obviously damaged in the septic mice (Figures 1D,E). The morphology of the ileal mucosal villi and the intestinal glands was severely disintegrated. In addition, the length of the villi in the intestinal tissues was significantly reduced in septic mice (Figure 1F). Both LSF and HSF effectively improved the morphologies of the intestine according to the Chiu’s pathological scores (Figures 1D,E). The length of the villi was also significantly increased by both LSF and HSF (Figure 1F). Additionally, Upon SFI administration, CLP-induced increased levels of lactate were significantly reduced (Figure 1G). These data indicated that both LSF and HSF have beneficial effects on the intestinal injury associated with sepsis.

### 3.2 Shenfu injection promoted intestinal regeneration in sepsis

**FIGURE 2:**
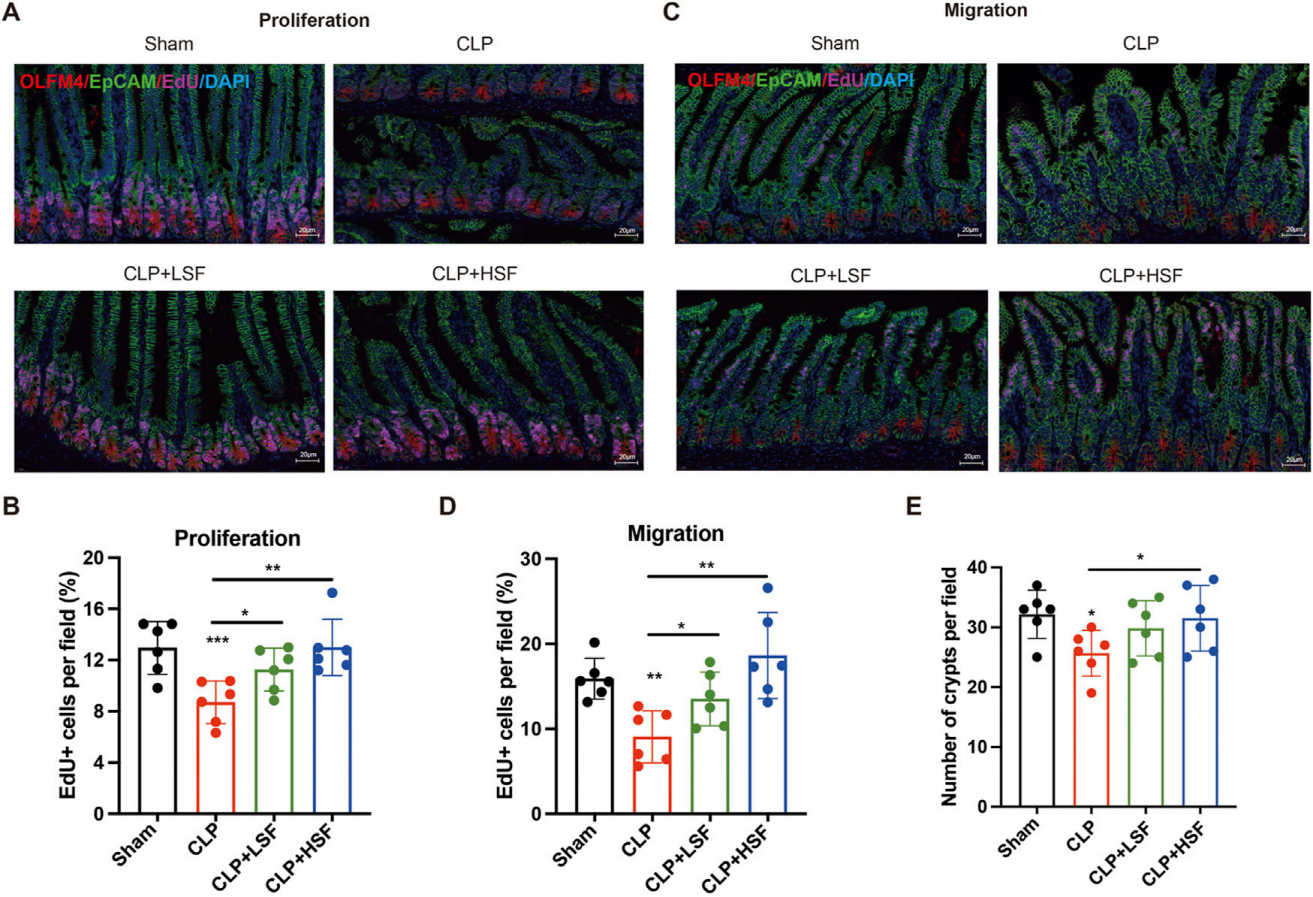
SFI treatment increased the proliferation of crypt/enterocytes and the migration of enterocytes in septic mice. **(A)** EdU was i.p. injected before mice were sacrificed. Crypt stem cells were stained with OLFM4 (red) and EdU were stained in pink. Nucleus were stained with DAPI (blue). [The white dash in the lower right corner of each picture represents 20 μm] **(B)** The EdU + cells in selected fields were blindly quantified by pathologists (n = 6 per group). **(C)** EdU was i.p. injected before mice were challenged with CLP. Enterocytes were stained with EpCAM (green) and EdU were stained in pink. Nucleus were stained with DAPI (blue). **(D)** The EdU + cells in selected fields were blindly quantified by pathologists (n = 6 per group). **(E)** The number of crypts in selected fields were blindly quantified by pathologists (n = 6 per group). *, P < 0.05, **, P < 0.01, ***, P < 0.001.

To investigate the effect of SFI on crypt proliferation, mice were i.p. injected with EdU before sacrifice. The results showed that the proliferation of the crypts was significantly decreased in septic mice, which was dramatically increased by both LSF and HSF (Figures 2A,B). Furthermore, to test the effect of SFI on the migration of enterocytes in the intestine, mice were i.p. injected with EdU before CLP challenge to allow EdU + cells to migrate for identical length of time along the villus. The results showed that sepsis-related decreases in the migration rate and distance of enterocytes were significantly increased by SFI (Figures 2C,D). Additionally, the number of the crypts in the intestine was significantly decreased in the septic mice and was restored by SFI administration (Figure 2E). These data indicated that SFI can promote crypt proliferation and enterocyte migration for the repair of villus damage induced by sepsis.

### 3.3 ATF4-mediated stemness activation involved in SFI-related increase of the proliferation of crypt stem cells

**FIGURE 3:**
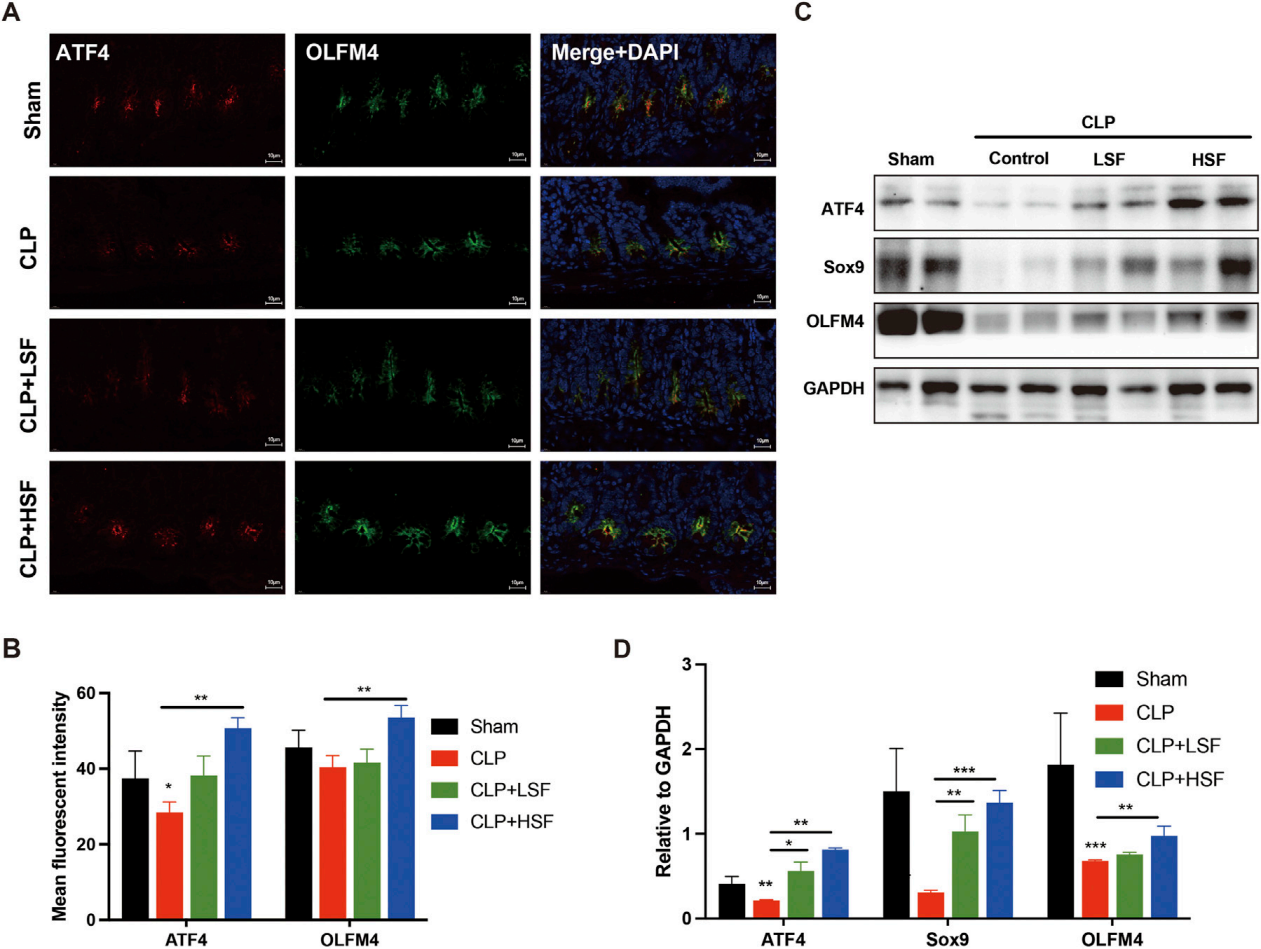
ATF4-mediated increase of the crypt stemness restored SFI-induced self-renewal of the intestine in sepsis. **(A,B)** Crypt stem cells in the intestinal tissues of different groups of mice were stained with OLFM4 (green) and ATF4 (red). The nuclei were stained with DAPI. [The white dash in the lower right corner of each A picture represents 10 μm] **(C,D)** The protein levels of ATF4, Sox9, and OLFM4 in the intestinal tissues were determined by Western blot assay. *, P < 0.05, **, P < 0.01, ***, P < 0.001.

ATF4 has been reported to positively modulate self-renewal maintenance of intestinal stem cells (Guo et al., 2023). It has been reported that ATF4 is associated with cell survival and proliferation (Williams et al., 2020). Therefore, we analyzed the expression of ATF4 and stemness-related markers, OLFM4 and Sox9, in the intestinal tissues of septic mice. The results showed that SFI significantly upregulated the expression of ATF4 both in OLFM4-stained crypt stem cells and intestinal tissues (Figures 3A,B). Additionally, the protein levels of ATF4, OLFM4, and Sox9 in the intestinal tissues of septic mice were significantly upregulated after HSF treatment (Figures 3C,D). These data imply that SFI-induced self-renewal of crypt stem cells might be mediated by ATF4-related activation of stemness, thereby alleviating sepsis-induced intestinal injury.

### 3.4 SFI increased the expression of tight junction proteins in the intestine

**FIGURE 4:**
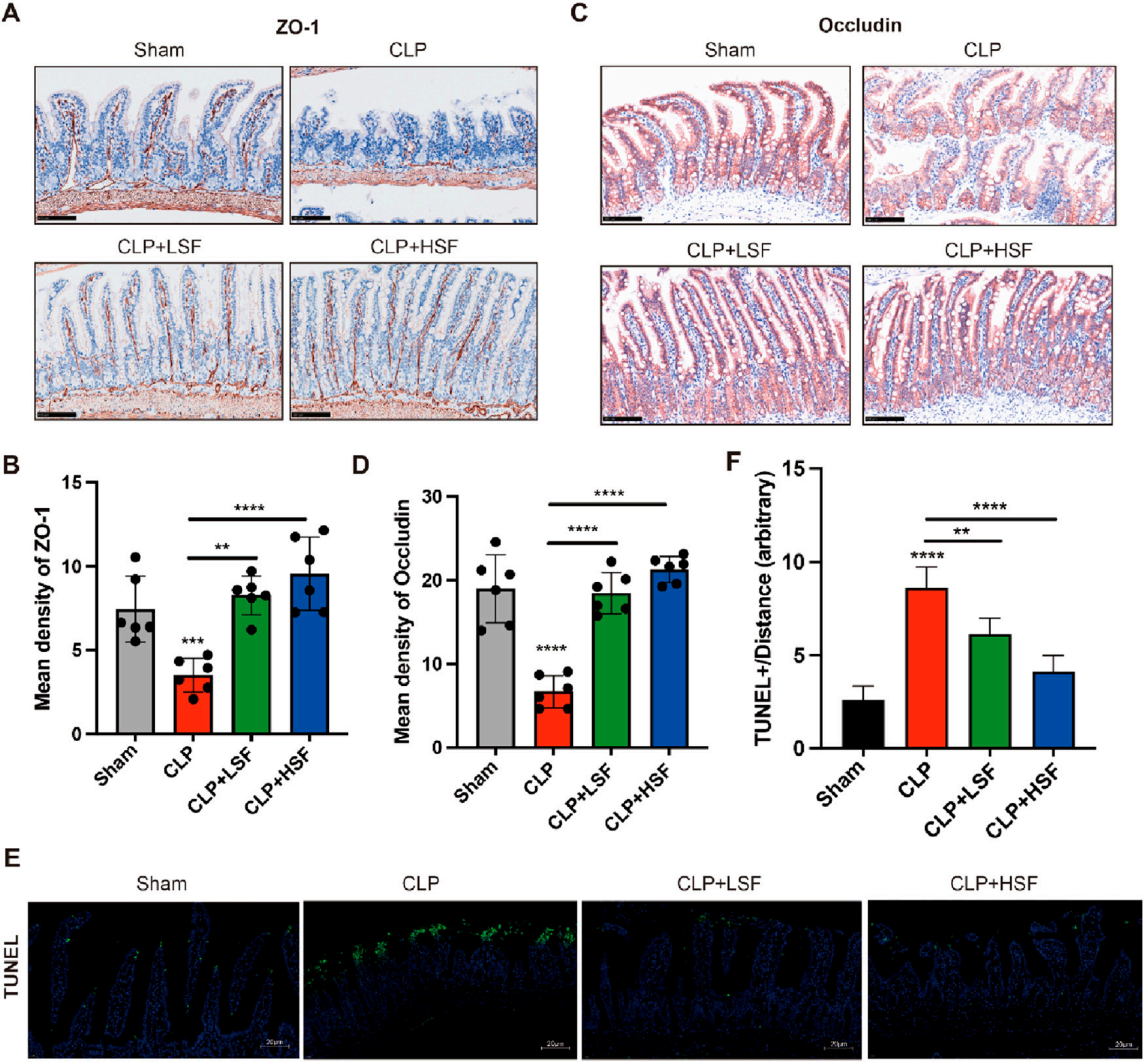
SFI treatment increased the expressions of tight junction proteins in the intestinal mucosa in septic mice. **(A,B)** IHC staining of ZO-1 in the intestinal tissues (n = 6 per group). **(C,D)** IHC staining of Occludin in the intestinal tissues. **(E,F)** The apoptosis in the intestinal tissue were determined by Tunnel staining. [The white dash in the lower right corner of each picture represents 20 μm] **, P < 0.01, ****, P < 0.0001.

The barrier function of the intestine is maintained by the expression of tight junction proteins (Marincola Smith et al., 2021; Wang J. et al., 2019). Therefore, we analyzed the effect of SFI on the expressions of tight junction proteins in the intestinal tissues of septic mice. The results showed that the expressions of ZO-1 and Occludin were significantly decreased in the intestinal tissues of septic mice, while LSF and HSF administration effectively upregulated ZO-1 and Occludin protein expressions in the mucosal villi (Figures 4A–D). Additionally, TUNEL staining showed that the apoptosis of the epithelial cells in the intestinal tissues of septic mice was significantly inhibited by SFI administration (Figures 4E,F). These data reveal the protective effects of SFI on the intestinal barrier function in sepsis.

### 3.5 Shenfu injection alleviated the inflammatory response in septic mice

**FIGURE 5:**
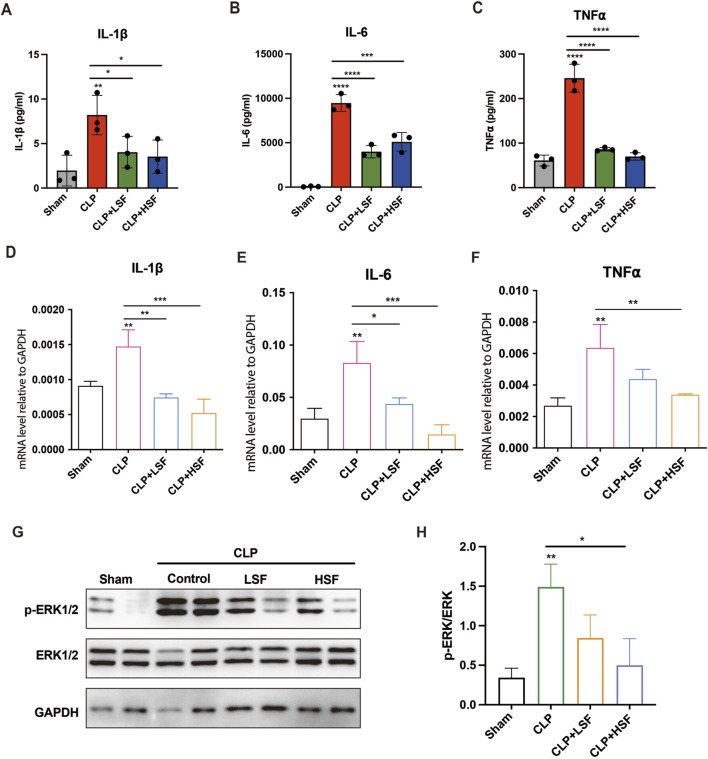
SFI alleviated the pro-inflammatory response in septic mice via inhibiting ERK1/2 phosphorylation. Mice were subjected to CLP before LSF and HSF administration. Mice were sacrificed after 16 h of CLP challenge. **(A–C)** The plasma levels of cytokines IL-1β, IL-6, and TNF-⍺ were measured by ELISA (n = 3). **(D–F)** The mRNA expressions of IL-1β, IL-6, and TNF-⍺ in the intestinal tissues were determined by RT-qPCR (n = 3). **(G,H)** The protein levels of phosphorylated ERK1/2 in the intestinal tissues were determined by Western blot. *, P < 0.05, **, P < 0.01, ***, P < 0.001, ****, P < 0.0001.

We further investigated the effects of SFI on systemic and local pro-inflammatory responses of septic mice. The plasma levels of Interleukin 1β (IL-1β), Interleukin 6 (IL-6), and tumor necrosis factor-⍺ (TNF-⍺) were significantly reduced by both LSF and HSF (Figures 5A–C). Additionally, the expression levels of pro-inflammatory cytokines in the intestinal tissues were significantly reduced by both LSF and HSF administration (Figures 5D–F). We then analyzed the effects of SFI on classical pro-inflammatory signaling pathways including mitogen-activated protein kinase (MAPK) signaling and nuclear factor kappa B (NF-κB) signaling. The results showed that the levels of phosphorylated ERK1/2 were significantly increased in the intestinal tissues of septic mice and were greatly inhibited by SFI treatment (Figures 5G,H). These data indicated that the activation of ERK1/2 signaling plays an important role in the anti-inflammatory effect of SFI on intestinal tissues during sepsis.

### 3.6 HSF exerted beneficial effects on gut microbiota in septic mice

**FIGURE 6:**
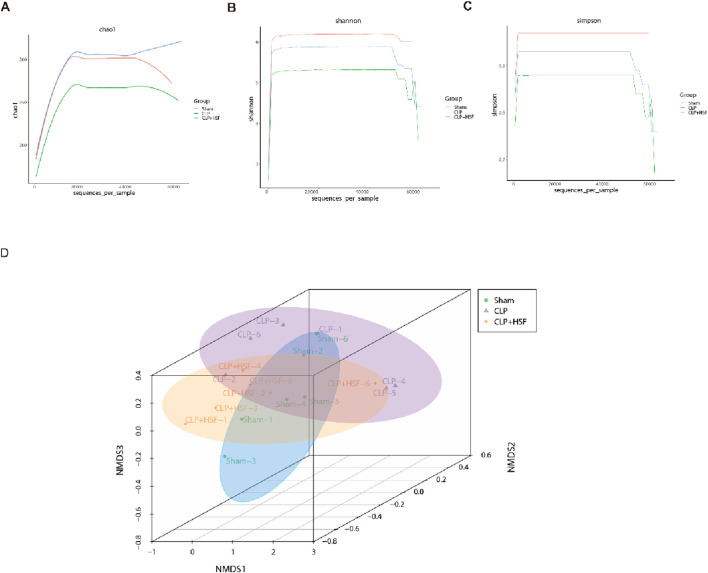
SFI administration modulated the diversity of the gut microbiota in septic mice. **(A–C)** The alpha diversity of gut microbiota including Chao1, Shannon, and Simpson indices. **(D)** The beta diversity of gut microbiota showing the compositions in different groups (n = 6 per group). *, P < 0.05, **, P < 0.01, ***, P < 0.001.

The gut microbiota is an important modulator of the host response to sepsis (Haak and Wiersinga, 2017). Therefore, we speculated that SFI alleviates intestinal injury by modulating the gut microbiota. 16S rRNA sequencing analysis was performed on cecum fecal samples to assess the composition of gut microbiota and the impact of SFI on the gut microbiota of septic mice. The results showed that the alpha diversity indices (Chao1, Shannon, and Simpson) of gut microbiota were reduced in septic mice compared to the control mice (Figures 6A–C). HSF restored the slight reduction in alpha diversity induced by sepsis (Figures 6A–C), suggesting that the alpha diversity of gut microbiota can be reversed by HSF treatment. We then examined the beta diversity of the gut microbiota to determine the variation in gut microbial composition among the groups. The gut microbial composition of the septic group was slightly different from that of the control group, whereas the gut microbial composition of the HSF group was more similar to that of the control group than that of the septic group (Figure 6D).

**FIGURE 7:**
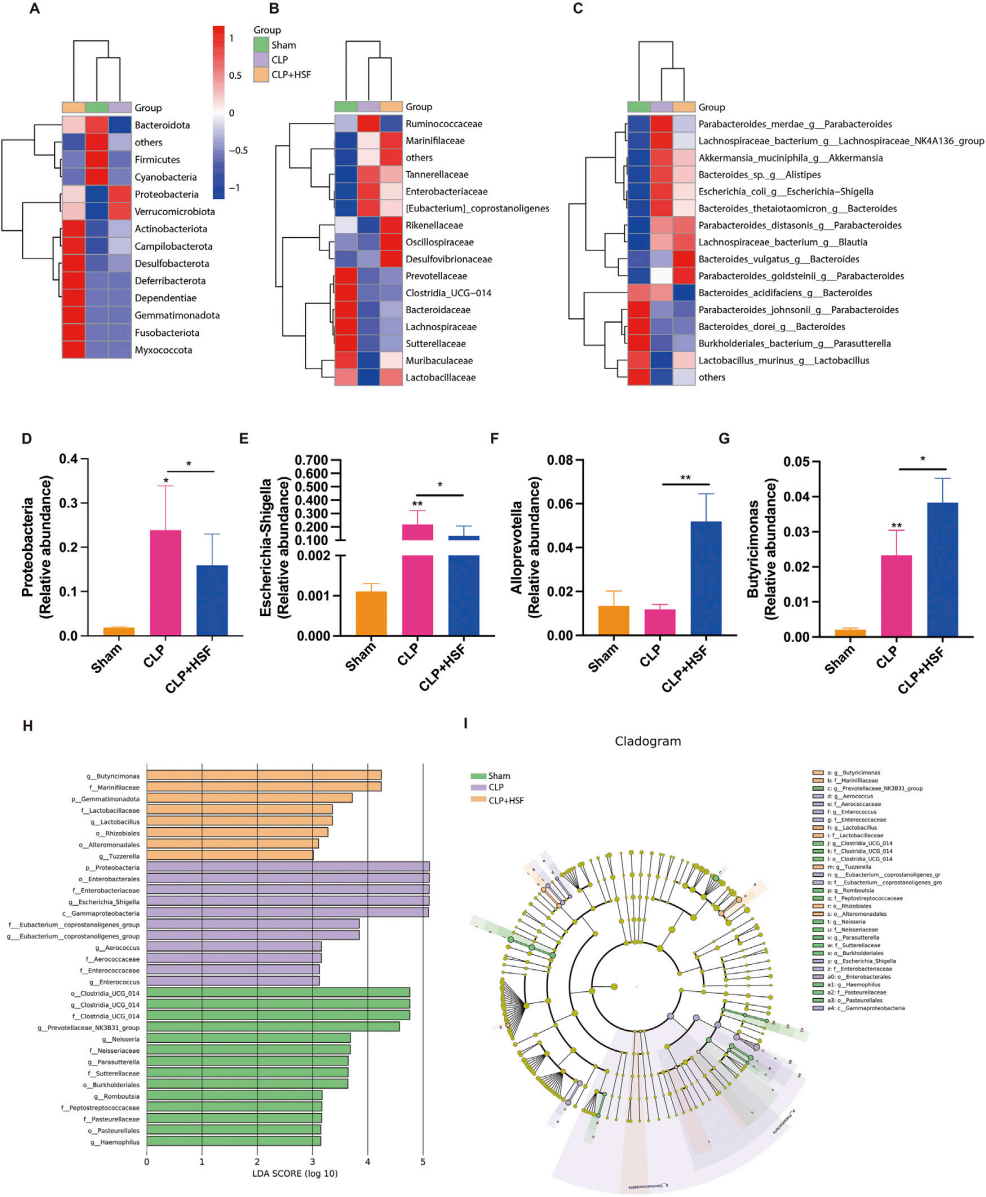
SFI altered the composition of gut microbiota in septic mice. **(A–C)** The gut microbiota showing differences among these groups at the phylum, family, and genus levels. **(D–G)** The abundance of proteobacteria, Escherichia-Shigella, Allpprevotella, and Butyricimonas in different groups (n = 6 per group). **(H,I)** LEfSe analysis of four groups. *, P < 0.05, **, P < 0.01.

Moreover, the gut microbiota exhibited differences between the control, septic, and SFI-treated groups at the phylum, family, and genus levels (Figures 7A–C). In particular, the relative abundance of Escherichia-Shigella and Proteobacteria which was related to lipopolysaccharide (LPS) production significantly increased in the septic group compared to the control group, which was slightly reduced by HSF administration (Figures 7D,E). In addition, the relative abundance of Alloprevotella and Butyricimonas, which are associated with the production of short chain fatty acids (SCFA) and the anti-inflammatory function, was slightly decreased in the septic mice and significantly increased in the high-dose SFI-treated group (Figures 7F,G). Linear discriminant analysis Effect Size (LEfSe) (LDA score (log10) > 4) was performed to identify the critical biomarkers of gut microbiota among various groups. In the septic group, Proteobacteria, Enterobacterales, Enterobacteriaceae, *Escherichia Shigella*, and Gammaproteobacteria was more abundant than the others. Additionally, in the high-dose SFI-treated group, Butyricimonas and marinifilaceae were more abundant compared to the others (Figures 7H,I). Collectively, these data revealed the regulatory importance of SFI in the gut microbiota during sepsis.

## 4 Discussion

Traditional Chinese Medicine (TCM) interprets sepsis through its unique theoretical framework, emphasizing a holistic perspective and syndrome differentiation-based treatment. In TCM, sepsis is regarded as the interplay between “pathogenic invasion” and “deficiency of vital Qi”. It posits that sepsis arises from imbalances in the body’s “Qi”, “Blood”, “Yin” and “Yang”, particularly the insufficiency of vital Qi, which allows external pathogens to invade, leading to compromised immune function, severe infection, and systemic inflammatory responses. SFI originated from the traditional Chinese formula “Shenfu Decoction”, which was first recorded in Ancient Chinese medical reference in the Song Dynasty (1253 AD). SFI is composed of Panax ginseng C.A.Mey and Aconitum carmichaelii Debeaux, which has the function of restoring Yang and invigorating Qi. Ginsenosides, the bioactive compounds in ginseng, have been confirmed the effect of inhibiting the production of proinflammatory cytokines and regulate inflammatory signaling pathways, such as NF-κB and activator protein-1, and exert anti-inflammatory effects during sepsis (Im, 2020).

The combination of ginseng with aconite is superior to single drug in stabilizing hemodynamics, enhancing cardiac function, and facilitating tissue tolerance to ischemia and hypoxia (Zhou et al., 2021; Wang et al., 2021). Meanwhile, ginsenosides could promote the metabolism of the toxic component aconitine, reduce toxicity and prolong the elimination half-life of active ingredients. The compatibility of aconite and ginseng has the effect of “reducing toxicity and increasing efficiency” (Xu et al., 2024).

The gut is the motor of MODS in sepsis (Klingensmith and Coopersmith, 2016). Sepsis-induced intestinal injury is characterized by the dysfunction of the mucosal barrier and disorder of the microbiome. Previous studies revealed alleviation of intestine epithelial damage in septic rats after SFI treatment (Xing et al., 2015; Hua et al., 2024). In this study, we found that SFI improved the renewal capacity of intestinal villi by promoting the proliferation of the crypts and the migration of the enterocytes, thereby alleviating sepsis-induced intestinal injury in mice. Mechanistically, ATF4 expression was induced by SFI in the intestinal crypts, thereby promoting the transcription of Sox9 in the stem cells, leading to increased proliferation of the crypt stem cells. Moreover, SFI exerts anti-inflammatory effect on intestinal tissues by inhibiting the phosphorylation of ERK1/2. In addition, the composition of the gut microbiota in septic mice treated with HSF switched from LPS synthesis and proinflammatory status to SCFA production and anti-inflammatory conditions. Our study supports the idea that SFI is beneficial for treating intestinal injury in sepsis.

Intestinal stem cells in the crypts play a pivotal role in maintaining the intestinal homeostasis. Sepsis induces growth arrest in intestinal crypt epithelial cells, suggesting decreased proliferation of stem cells in the intestines due to severe acute inflammation (Geng et al., 2018). We have previously shown that sepsis decreases crypt proliferation, the enterocyte migration rate, and migration distance in the villus (Meng et al., 2019). Cell migration is associated with cell proliferation in the small intestine. In previous researches, as an important bioactive constituent of total ginsenosides, Ginsenoside Rg1 has demonstrated beneficial effects on intestinal health (Cheng et al., 2022; Yousuf et al., 2022). In our study, injection of EdU at the end of the *in vivo* study labeled cells in the S-phase, reflecting the status of cell proliferation after SFI treatment and CLP challenge. However, EdU injection before sepsis labeled cells with normal proliferation and allowed EdU-intercalated cells to migrate along the villus during SFI treatment and CLP challenge. We found for the first time that SFI administration significantly increased crypt/enterocyte proliferation and migration in septic mice.

SFI is clinically used for its antishock pharmacological effects. Previous studies have reported that SFI treatment can prevent sepsis-induced myocardial dysfunction by inhibiting mitochondrial and cardiomyocyte apoptosis (Xu et al., 2020; Yan et al., 2018). SFI can also suppress the blood levels of cytokines and improve the vital signs of patients with severe pneumonia (Lv et al., 2017). Recently, it has been reported that SFI can improve microvascular dysfunction and clinical outcomes in patients with septic shock, cardiac arrest, as well as gastrointestinal microcirculation after myocardial ischemia-reperfusion injury (Wang et al., 2022; Shao et al., 2020; Zhang et al., 2006). Moreover, SFI improves the mucosal mechanical barrier and inhibits intestinal permeability, thereby preventing sepsis-related intestinal injury (Wu et al., 2015; Liu et al., 2021). In the present study, we found beneficial effects of SFI on the intestinal integrity during sepsis by increasing the crypt proliferation and the enterocyte migration. In light of these findings, it is of interest to explore the therapeutic efficacy of SFI on other organ injuries associated with sepsis, thereby expanding the clinical application of SFI for the treatment of sepsis-related MODS.

The gut microbiome plays a pivotal role in the progression of sepsis. Herbal medicine has the potential to improve the disorders of the intestinal microbiota and regulate intestinal function. Zong’s study revealed protection effects of Ginsenoside Rb1 on intestinal stem cells either through the intestinal microbiota or through microbiota-associated metabolites (Zong et al., 2024). To our knowledge, this is the first study to elucidate the effect of SFI on gut microbiota in septic mice. Alpha diversity analysis showed that the richness of gut microbiota was slightly decreased in septic mice, and the same conclusion has been reported in acute ulcerative colitis mice (Cheng et al., 2022; Su et al., 2020). Administration of HSF restored the slight reduction of alpha diversity induced by sepsis. Consistently, Ginsenoside Rg1, as the main active constituent of SFI, has been shown to reverse the decreased richness of the gut microbiota caused by dextran sulfate sodium (DSS) challenge (Cheng et al., 2022). Additionally, NMDS analysis showed that the groups treated with HSF treatment were closer to the control group compared to CLP-challenged group. Sepsis can induce a relative abundance of potentially harmful bacteria, and the administration of HSF can partly mitigate the effects of sepsis. Proteobacteria are pathogenic bacteria that includes *Pseudomonas aeruginosa* and *Pseudomonas* putida, which have been reported to cause severe pneumonia in critically illness and immunocompromised patients (Wu et al., 2021). The prevalence of Proteobacteria is a potential diagnostic signature of the disease (Shin et al., 2015). In our study, the dramatically increased Proteobacteria upon the challenge with sepsis at the phylum level was slightly decreased by HSF. At the genus level, the increased level of pathogens associated with proinflammatory response, including Escherichia-Shigella and *klebsiella*, destroyed the gut microecological balance and increased inflammation. Escherichia-Shigella has been reported to be associated with obesity-related metabolic dysfunction (Kong et al., 2019). Moreover, a HSF treatment increased the abundance of Alloprevotella and Butyricimonas which produce SCFA to provide energy for intestinal cells, maintain the intestinal barrier (Koh et al., 2016), and prevent LPS translocation from the intestinal barrier (Zhou et al., 2017; Kelly et al., 2015). In addition, SCFA has also been reported to play an indispensable role in regulating immune homeostasis (Feng et al., 2018; Blaak et al., 2020). These results suggest that the suppression of pathogenic bacteria and the increase in beneficial bacteria by HSF may be the mechanism of SFI in maintaining intestinal integrity in sepsis.

Our study has some limitations. First, the clinical sample size might have been insufficient to detect a clinically meaningful treatment effect. Considering that this research was mainly focused on animal experiments, and the clinical data was used as a supporting part, there was no large-scale investigation in terms of clinical data collection. Second, there is no clear causal relationship between microbiota changes and intestinal function status, but we observed this phenomenon in our study, which might provide direction for future research. Third, the critical role of ATF4 in SFI’s effects lacks direct evidence, and the detailed pathway is not quite clear. The specific mechanism needs to be confirmed by more research.

## 5 Conclusion

Our study demonstrated that HSF represents a promising strategy for treating sepsis-induced intestinal injury, since it can promote ATF4-mediated self-regeneration of the intestine by increasing the crypt/enterocyte proliferation and migration. Additionally, SFI inhibits ERK1/2 phosphorylation, thereby alleviating the pro-inflammatory response in the intestine during sepsis. Moreover, HSF slightly decreased the sepsis-induced elevation of Proteobacteria and Escherichia-Shigella and increase the abundance of Alloprevotella and Butyricimonas in the gut. These findings provide novel insights into the clinical application of SFI in sepsis.

## Data Availability

The data presented in the study are deposited in the NCBI repository, accession number PRJNA1294597.

## Abbreviations

ATF4, activating transcription factor 4; CLP, cecal ligation and puncturece; Cy3, Cyanine3; DAO, diamine oxidase; EDTA, Ethylenediaminetetraacetic acid; EdU, 5-Ethynyl-2′- deoxyuridine; ELISA, enzyme-linked immunosorbent assay; EpCAM, Epithelial cellular adhesion molecule; ERK1/2, extracellular signal-regulated kinases 1 and 2; H&E, hematoxylin-eosin; HRP, horseradish peroxidase; HSF, high dose of Shenfu injection; IHC, immunohistochemistry; IL-1β, Interleukin 1β; IL-6, Interleukin 6; LEfSe, Linear discriminant analysis Effect Size; LSF, low dose of Shenfu injection; LPS, lipopolysaccharide; MODS, multiple organ dysfunction syndrome; NF-κB, nuclear factor kappa B; NMDS, Non-metric Multidimensional Scaling; OLFM4, Olfactomedin 4; RT-qPCR, quantitative reverse transcription-PCR; SCFA, short chain fatty acid; SFI, Shenfu injection; Sox9, SRY-Box Transcription Factor 9; TNF-⍺, tumor necrosis factor-⍺; TCM, Traditional Chinese Medicine; ZO-1, Zonula Occludens-1.
