# Supplementary material for: Shenfu injection promotes self-renewal of intestinal stem cells in sepsis-induced intestinal injury via inducing ATF4 expression
Source: Front Pharmacol. 2025 Jul 31;16:1500157. doi: 10.3389/fphar.2025.1500157 (PMC12351133; doi:10.3389/fphar.2025.1500157)
Supplement: Supplementary file 1 [file Table1.docx]

**Supplementary Table 1. Basline demographic, clinical, and follow-up characteristics of the study patients.**

| Characteristics | All patients  N=41 | SFI  N=20 | Non-SFI  N=21 | *P* value |
| --- | --- | --- | --- | --- |
| Median age (IQR), yr | 64.00（53.25-76.00） | 64.00（53.00-76.00） | 63.50（57.75-74.00） | 0.587 |
| Gender, n (%) |  |  |  |  |
| Female | 14（34.15） | 6（31.25） | 8（38.10） | 0.786 |
| Male | 27（65.85） | 14（70.00） | 13（61.90） |  |
| Comorbidity |  |  |  |  |
| Diabetes, n (%) | 10（24.40） | 5（25.00） | 5（23.81） | 0.37 |
| Hypertension, n (%) | 8（19.51） | 4（20.00） | 4（19.05） | 0.21 |
| Coronary heart disease, n (%) | 8（19.51） | 4（20.00） | 4（19.05） | 0.03 |
| COPD, n (%) | 7（17.07） | 3（15.00） | 4（19.05） | 0.03 |
| Cancer, n (%) | 4（9.8） | 2（10.00） | 2（9.52） | 0.76 |
| Primary site of infection, n (%) |  |  |  |  |
| Pulmonary | 9 (21.95) | 5 (25.00) | 4 (19.05) | 0.934 |
| Gastrointestinal or intra-abdominal | 26 (63.41) | 13 (65.00) | 13 (61.90) | 0.837 |
| Blood | 1 (2.44) | 1 (5.00) | 0 (0.00) | 0.488 |
| Other | 5 (12.20) | 1 (5.00) | 4 (19.05) | 0.370 |
| APACHE II score at ICU admission | 16.00 (8.00, 22.00) | 19.00 (8.75, 21.00) | 11.00 (7.00, 22.00) | 0.834 |
| SOFA score at ICU admission | 5.00 (3.00, 8.00) | 5.50 (3.00, 7.00) | 5.00 (3.00, 9.00) | 0.486 |
| Type of ICU admission |  |  |  |  |
| Non-surgical emergency | 10 (24.39) | 5 (25.00) | 5 (23.81) | 1.000 |
| Surgical emergency | 15 (36.59) | 9 (45.00) | 6 (28.57) | 0.275 |
| Others | 16 (39.02) | 6 (30.00) | 10 (47.62) | 0.248 |
| Treatment during hospitalization |  |  |  |  |
| Invasive mechanical ventilation treatment, n (%) | 20 (48.78) | 9 (45.00) | 11 (52.38) | 0.636 |
| Continuous renal replacement therapy treatment, n (%) | 33 (80.49) | 15 (75.00) | 18 (85.71) | 0.638 |
| ICU LOS, days | 41.00 (26.00, 93.00) | 34.00 (23.50, 82.50) | 50.00 (31.00, 101.00) | 0.375 |
| Hospital LOS, days | 46.00 (28.00, 101.00) | 37.50 (27.00, 85.00) | 50.00 (37.00, 101.00) | 0.557 |
| ICU mortality | 10 (24.39) | 4 (20.00) | 6 (28.57) | 0.783 |
| Hospital mortality | 20 (48.78) | 9 (45.00) | 11 (52.38) | 0.636 |
